# Effects of Continuous Positive Airway Pressure for Hypertension in Patients with Obstructive Sleep Apnoea: A Structured Narrative Review of Randomised Controlled Trials

**DOI:** 10.3390/jcm15124475

**Published:** 2026-06-09

**Authors:** Ashwag Alsharidah

**Affiliations:** Department of Physiology, College of Medicine, Qassim University, Buraydah 52571, Saudi Arabia; ashriedt@qu.edu.sa

**Keywords:** continuous positive airway pressure, structured narrative review, randomised controlled trials, obstructive sleep apnoea, systolic blood pressure, diastolic blood pressure

## Abstract

**Introduction**: Obstructive sleep apnoea usually co-occurs with hypertension and is managed using various therapeutic modalities. Continuous positive airway pressure (CPAP) appears to be one of the promising interventions. This review aims to narratively assess its effectiveness by comparing its effect with usual and standard care. **Method**: The Preferred Reporting Items for Systematic Reviews and Meta-Analyses (PRISMA) 2020 guidelines were consulted to enhance transparency in reporting the search and selection process; however, this review does not meet the full criteria for a systematic review because screening, data extraction, and synthesis were not conducted according to full systematic review methodology. Five electronic databases (APA PsycINFO, Web of Science, Embase, AMED, and Medline) were searched. Only randomised controlled trials were considered eligible and were assessed for risk of bias using the Physiotherapy Evidence Database (PEDro) scale. Effect sizes were calculated using an online tool developed by the Campbell Collaboration, George Mason University, version 27 November 2023. They were interpreted as trivial (<0.1), small (0.1–0.3), moderate (0.3–0.5), or large (>0.5) effects, respectively. **Results**: This study includes 2944 subjects, 63.3% of whom were male. Their ages range from 23 to 69.7 years. The effect sizes for systolic blood pressure ranged from small to large (d = −0.2, 95% CI −0.69 to 0.28, to d = 0.7, 95% CI −0.15 to 1.55), and for diastolic blood pressure from d = 0.1, 95% CI −0.3 to 0.57, to d = 1.4, 95% CI −0.97 to 2.08 magnitude of effects was observed for both systolic and diastolic blood pressure at different times of the day. Standard care, such as medication and telemedicine, does not confer superior effects over usual care. This intervention showed evidence of sustained effects; however, further evidence is required. **Conclusions**: CPAP may be effective for both systolic and diastolic blood pressure in patients with obstructive sleep apnoea at different times of day. Its effect may be sustained. The effective dose of CPAP on hypertension remains unclear. Future studies should consider using a more robust design, such as a systematic review and meta-analysis, to obtain pooled estimates rather than individual effect sizes.

## 1. Introduction

Obstructive sleep apnoea (OSA) is a sleep disorder defined as intermittent sleep state-dependent upper airway failure, resulting in episodic reductions or terminations in breathing [1]. OSA is a significant risk factor for cardiovascular disease [2]. Literary evidence has shown that OSA may influence the development of cardiovascular problems, particularly by activating pathways leading to oxidative stress, impaired sympathetic drive, overactivity, inflammation, hypercoagulability, and endothelial dysfunction, thus resulting in conditions such as atherosclerosis, hypertension, heart failure, and stroke [1,3]. OSA and hypertension frequently co-exist, with 30–50% of hypertensive patients having OSA. Furthermore, 30–70% of OSA patients have been reported to have hypertension, and it may be the most prevalent contributor to resistant hypertension [4,5,6].

**Figure 1:**
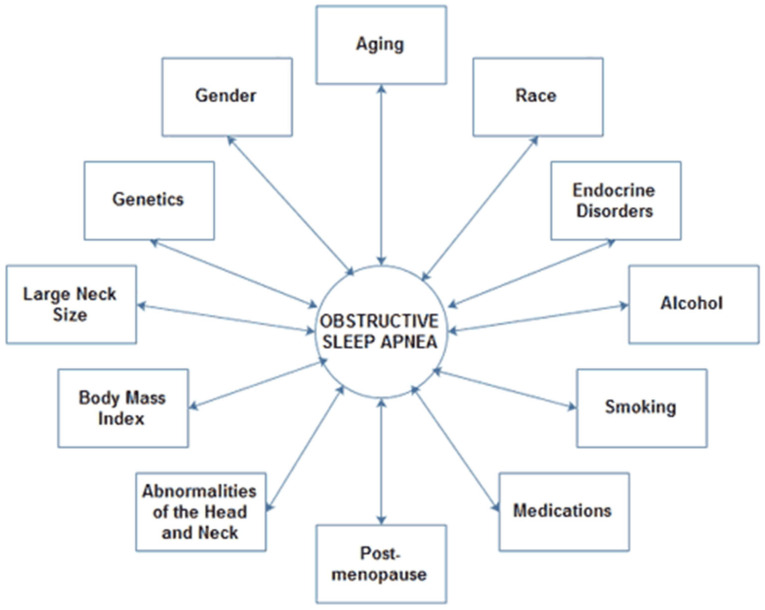
Widely Recognised Risk factors for OSA. Sources: https://www.sleep-apnea-guide.com/causes-of-sleep-apnea.html (Accessed 15 April 2026).

As shown in Figure 1, OSA has many risk factors, including body mass index (BMI), particularly for those with BMI ≥ 30; large neck size (≥17 inches in men and ≥16 inches in women); family history (genetics); menopause (3–5 times higher in postmenopausal than in premenopausal women); ageing (≥65 years); race (African American); endocrine disorders (hypothyroidism, macroglossia, and acromegaly); alcohol use (relaxes upper airway muscles); smoking (reduces oxygen levels); medications (hypnotics and sedatives); and abnormalities of the neck and soft tissues [7,8].

Another important factor is gender, as evidence has shown that OSA may be 2–3 times more common in men than in women [9]. OSA is a sex-specific cardiovascular risk factor, particularly because it is underdiagnosed in women [10]. OSA often remains undiagnosed and undertreated in women, partly because screening tools and clinical criteria have been developed mainly in male cohorts [9,10].

The primary treatment for OSA is positive airway pressure (PAP) therapy, which includes CPAP, Auto-setting Positive Airway Pressure (APAP), and Bilevel Positive Airway Pressure (BiPAP) [11,12]. Oral appliance therapy (OAT), such as mandibular advancement devices (MADs) and tongue-retaining devices, is also used, as are surgical interventions (e.g., upper airway stimulation) [13,14]. Other options include behavioural and lifestyle changes, such as weight loss, positional therapy, and avoidance of certain substances [15]. The literature also indicates that oropharyngeal exercises and expiratory positive airway pressure may be beneficial [16,17]. Surgery is also an option, including nasal surgery and maxillomandibular advancement, which may be considered when other strategies fail [18]. This approach is considered secondary and may serve as a salvage therapy, particularly for adults who cannot tolerate CPAP or any known oral appliances [18]. Another effective treatment for OSA, particularly for obese individuals, is bariatric surgery, as evidence has shown that it may result in significant weight reduction or resolve OSA in 61.4% and 52.6% of patients [19]. The weight reduction, in particular, was considered a long-term outcome of CPAP use for bariatric patients; in contrast, non-adherence was known to be associated with significant weight gain, and its hindrance might potentially interfere with long-term weight management [20].

The mechanism for the development of hypertension was said to be associated with activation of the sympathetic nervous system and the influence of the renin–angiotensin–aldosterone system, which might be associated with impairment of endothelial function [21].

Several studies focus on the impact of sleep apnoea on the sympathetic nervous system and cardiovascular health [22,23], heart failure [24], diagnostic biomarkers for coronary artery disease and OSA [25], and the association between triglyceride–glucose index and the risk of cardiovascular diseases [26]. Furthermore, OSA and its relationship with cardiovascular diseases, including hypertension, have been widely explored using observational and prospective studies, and evidence has shown a positive association with hypertension [21,27]. Specifically, the available review examining the updated association between OSA and hypertension was published in 2017. It does not explore specifically the changes that take place in subjects’ blood pressure; rather, it focuses on correlations and multimodal treatment [28,29]. It is important to note that these studies were published 8–9 years ago; therefore, an update may be necessary. Furthermore, to date, there appears to be no structured narrative review of randomised controlled trials that specifically examines the effects of CPAP on hypertension in patients with obstructive sleep apnoea, particularly focusing on different timings of the day. This study aims to address this gap.

### Review Objective

1. To identify the effects of CPAP on hypertension in patients with obstructive sleep apnoea.
2. To determine the effective dose of CPAP on systolic and diastolic blood pressure outcomes.

## 2. Methods

The PRISMA 2020 guidelines were consulted to enhance transparency in reporting the search and selection process; however, this review does not meet the full criteria for a systematic review because screening, data extraction, and synthesis were not conducted according to full systematic review methodology [30].

### 2.1. Eligibility Criteria

The inclusion criteria are: Population: (1) adults aged ≥18 years, and (2) diagnosis of obstructive sleep apnoea (OSA) by either home sleep apnoea testing or polysomnography, with comorbid hypertension, (3) studies must be randomised controlled trials, and (4) interventions: must be CPAP that was compared against usual or standard care. We will exclude studies that were not experimental in design, as well as case studies, letters to the editor, protocols, conference proceedings, theses, or dissertations.

### 2.2. Information Sources

Five electronic databases will be searched via the Saudi Digital Library (SDL): (1) Embase, (2) Medline, (3) Web of Science (WoS), (4) PsycINFO, and (5) AMED. The search will be conducted without restriction on date or time of publication; however, only papers with full text and abstract available, published in English, will be considered. A manual search for the grey literature will be conducted using the first ten pages of Google Scholar.

### 2.3. Search Strategy

The search terms were developed using the MeSH on Demand of the National Library of Medicine. We used the Boolean operators of “AND” **Population or problem**: Obstructive sleep apnoea OR OSA OR Sleep Apnoea OR Obstructive AND **Outcome**: Hypertension OR High Blood pressure OR Homeostatic changes OR Homeostasis OR Neuronal plasticity AND **Study Design**: Quasi-experimental OR Randomised Controlled Trial OR RCT OR Pre-test post-test design.

### 2.4. Selection Process

The search results from the five electronic databases will be imported into the reference manager (EndNote, version 20) for deduplication. This will be followed by screening titles and abstracts against the inclusion and exclusion criteria. In addition, only one person (the researcher) screens the papers in full text. This was conducted by a single reviewer, and the absence of a second reviewer may present a significant limitation for this review.

### 2.5. Data Collection Process

A Microsoft Excel spreadsheet will be developed to extract data. The author independently extracts the data without the involvement of a third neutral party.

### 2.6. Data Items

Data extracted were the study design, author and year, study population, sample size, outcomes (their means (M), standard deviations (SD), and their respective measures).

### 2.7. Risk of Bias Assessment

The methodological quality of the included RCTs was assessed using the PEDro scale, which has demonstrated validity as a measure of trial quality [31]. Although originally developed for physiotherapy research, its criteria are applicable to randomised trials across clinical medicine, including random allocation, concealed allocation, baseline comparability, blinding, attrition, intention-to-treat analysis, between-group comparisons, and reporting of variability. Therefore, we used the PEDro scale. This scale was developed for assessing the quality of RCT [31]. Evidence has shown that this tool has excellent psychometric properties [31]. It could provide information on the external and internal validity, as well as the statistical reporting subscales [31]. Rating was recorded as either “YES” or “NO” to indicate the presence or absence of an item. To obtain the PEDro total score, items 2 to 11 were summed; items 2 to 9 provide the internal validity subscale score, while items 10 and 11 are for the statistical reporting subscale score [31]. The PEDro total score may reflect the methodological quality of an RCT [31]. The interpretations were rated as follows: 0–3 for poor, 4–5 for moderate, 6–8 for good, and 9–10 for excellent quality [32,33].

### 2.8. Summary of Measures and Synthesis of Results

For effective guidance, refer to the Cochrane Handbook of Systematic Reviews and Meta-Analysis [34]. For the relevant outcomes indicating homeostatic changes, we will extract the mean (M) and standard deviation (SD) for both the experimental and control arms at baseline (T0) and post-intervention (T1). These data were extracted from all eligible studies, with attention to the time of day (morning, afternoon, evening, nighttime (nocturnal), or daytime (diurnal). Between-group effect size (Cohen’s d) was calculated using the aforementioned pre-post SD with a pre-post correlation of 0.5. An online effect size tool developed by the Campbell Collaboration, George Mason University, version date: 27 November 2023, available at: https://www.campbellcollaboration.org/calculator/d-means-sds-with-pretest (Accessed 23 February 2026) was used to estimate the effects between the two study arms. Effect sizes were classified as trivial (<0.1), small (0.1–0.3), moderate (0.3–0.5), and large (>0.5), with their corresponding confidence interval (CI) [35].

## 3. Results

**Figure 2:**
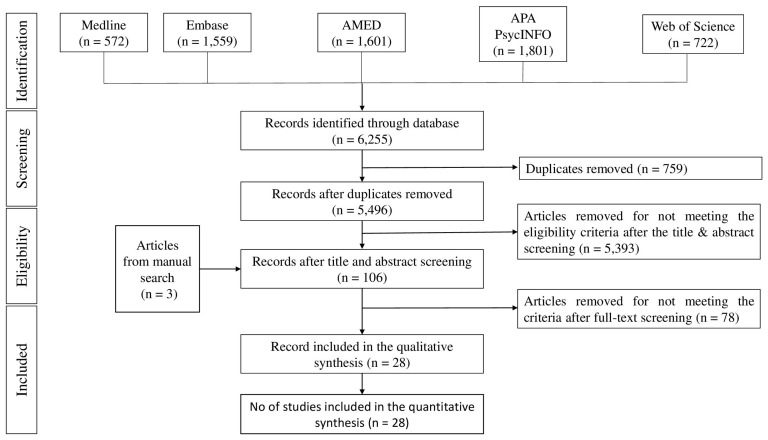
PRISMA flow chart.

As shown in Figure 2, a total of 6255 results were generated from the five electronic databases (Medline *n* = 573, Embase *n* = 1559, AMED *n* = 1601, APA PsycINFO *n* = 1801, and Web of Science *n* = 722). After duplicates were removed, 5496 records remained. These records underwent title and abstract screening, resulting in the exclusion of 5393 papers. Furthermore, three papers were added from the manual search. Thus, 106 papers were subjected to full-text screening. After full-text screenings, twenty-eight papers were included in the quantitative and qualitative syntheses.

### 3.1. Risk of Bias

As shown in Table 1, most studies (*n* = 22, 78.6%) presented their eligibility criteria, and all studies randomly allocated their subjects, compared the two groups, and provided point measures and variability data. The dropout rate was low, with most studies (*n* = 16, 57.1%) having less than 15%. Similarly, most studies (*n* = 15, 53.6%) treated their research subjects using intention-to-treat approaches. However, the majority of studies (*n* = 22, 78.6%) and (*n* = 15, 53.6%) did not blind the participants and assessors, respectively. Uniquely, only one study blinded the therapist, while the majority (*n* = 27, 96.4%) did not. The quality of the studies ranges from good (*n* = 17, 60.7%) and moderate (*n* = 10, 35.7%) to excellent (*n* = 1, 3.6%).

### 3.2. Demographic Characteristics

As shown in Table 2, there were 2944 subjects in total, with males (*n* = 1863, 63.3%) constituting the majority. Their mean age ranged from 23 to 69.7 years. Most studies (*n* = 19, 67.9%) were conducted in hospitals. The studies were published between 2004 and 2025. The interventions compare CPAP with sham CPAP, no CPAP, antihypertensive medication, telemedicine-supported care, APAP, and nasal dilator strips, making them heterogeneous.

### 3.3. Effects of Continuous Positive Airway Pressure on Hypertension in Patients with Obstructive Sleep Apnoea

#### 3.3.1. Continuous Positive Airway Pressure for Systolic and Diastolic Blood Pressure

##### Effects of Continuous Positive Airway Pressure Versus Usual Care on Systolic Blood Pressure

It was reported that there was no significant change for the CPAP: −3.6, 95% CI: −18.45 to 11.25, *p* = 0.50 [38]. Specifically, moderate effects were observed (−0.5, 95% CI: −0.98 to −0.11) [56], as well as large effects (d = −0.6, 95% CI: −1.17; d = −0.11; −0.7, 95% CI: −1.17 to −0.26) [58,64]. Further evidence has shown that CPAP significantly reduces systolic blood pressure, with an intergroup difference of −4.4 mmHg (95% CI: −8.7 to −0.1, *p* = 0.046) [59]. A small effect (d = −0.2, 95% CI: −0.64, 0.30) was also reported [46]. A trivial effect (d = −0.004, 95% CI: −0.37 to 0.36) was reported [57]. This effect appears to be similar to that for mean blood pressure (d = −0.009, 95% CI: −0.45 to 0.15) (Table 3) [62].

##### Effects of Continuous Positive Airway Pressure Versus Standard Care on Systolic Blood Pressure

The 24 h mean blood pressure reportedly showed effects ranging from small (d = −0.2, 95% CI: −0.69, 0.28; d = 0.1, 95% CI: −0.11, 0.34) to large (d = 0.7, 95% CI: −0.15, 1.55; d = −1.0, 95% CI: −1.38, −0.57) [36,51,61,62]. In contrast, the 24 h systolic blood pressure had a trivial effect (d = 0.06, 95% CI: −0.43, 0.54; d = 0.08, 95% CI: −0.13, 0.29) [36,65]. For systolic blood pressure at 3–12 months, effects ranged from trivial (d = 0.02, 95% CI: −0.19, 0.22; d = 0.08, 95% CI −0.13, 0.29; d = 0.02, 95% CI −0.19, 0.22) to small (d = 0.1, 95% CI: −0.07, 0.35; d = 0.1, 95% CI: 0.07, 0.35) [65]. However, when comparing the intervention with medication, a large effect was observed (d = −0.8, 95% CI: −0.72, 2.27) (Table 3) [40,42].

##### Effects of Continuous Positive Airway Pressure Versus Usual Care on Diastolic Blood Pressure

A small improvement was reported with an effect size of d = 0.1 (95% CI: −0.36 to 0.57) [46]. In contrast, a large effect was observed (d = −1.4, 95% CI: −2.08 to −0.97) (Table 3) [64].

##### Effects of Continuous Positive Airway Pressure Versus Standard Care for Diastolic Blood Pressure

The 24 h diastolic blood pressure showed a trivial effect (d = 0.07, 95% CI: −0.41, 0.56) to a large effect (d = 0.8, 95% CI: −0.09, 1.63) [36,61]. For changes in diastolic blood pressure, a small effect (d = −0.2, 95% CI: −0.37 to 0.04; d = −0.2, 95% CI: −0.41, 0.04), moderate (d = −0.5, 95% CI: −0.88, −0.14) to large (d = −0.7, 95% CI: −1.60, 0.25) effects were reported [40,51,62,65]. Furthermore, a small effect size was observed in six months (d = 0.1, 95% CI: −0.09, 0.33) and twelve months (d = 0.1, 95% CI: −0.07, 0.35), respectively (Table 3) [65]. Similarly, for 3–12 months, a small change in diastolic blood pressure was reported, with effect sizes of d = 0.2, 95% CI: −0.04, 0.37; d = 0.2, 95% CI: 0.03, 0.45; and d = 0.3, 95% CI: 0.05, 0.47, respectively (Table 3) [42].

##### Effects of Continuous Positive Airway Pressure Versus Usual Care on Systolic Blood Pressure at Specific Times of Day

For the specific timings, small (d = −0.2, 95% CI: −0.89, 0.45) to moderate effects (d = −0.4, 95% CI: −1.11, 0.26) were observed in the morning for both central and peripheral pressures [45]. Similarly, small (d = −0.2, 95% CI: −0.85, 0.48) to moderate effects (d = −0.4, 95% CI: −1.04, 0.32) for central and peripheral pressures were also reported in the afternoon [45]. For daytime systolic blood pressure, effects ranged from trivial (d = 0.04, 95% CI: −0.35, 0.42; 0.07, 95% CI: −0.24, 0.38; 0.008, 95% CI: −0.38, 0.40; 0, 95% CI: −0.31, 0.31) to large (d = −0.6, 95% CI: −1.24, 0.01) [47,58,60]. Similarly, for nighttime systolic blood pressure, effects ranged from trivial (d = −0.03, 95% CI: −0.42, 0.35; d = −0.03, 95% CI: −0.42, 0.35) to small (d = −0.3, 95% CI: −0.85, 0.34; −0.07, 95% CI: −0.38, 0.23; d = −0.2, 95% CI: −0.49, 0.13) and large (d = −0.8, 95% CI: −1.27, −0.35) (Table 3) [47,56,58,60].

Furthermore, small (d = −0.3, 95% CI: −0.50, 0.06) to moderate (d = −0.4, 95% CI: −0.57, −0.13) effects were observed for nighttime systolic blood pressure in an intervention lasting 6–12 weeks [39]. A similar pattern of changes with a small effect size was reported for daytime systolic blood pressure at six weeks (d = −0.2, 95% CI: −0.46, −0.03) and twelve weeks (d = −0.2, 95% CI: −0.38, 0.05). Finally, the 24 h daytime systolic blood pressure showed a small effect (d = −0.2, 95% CI: −0.59, 0.25) (Table 3) [56]. Additionally, one study reported a mean difference for daytime systolic blood pressure (−0.1, 95% CI: −2.4, 2.1) (Table 3) [54]. Daytime systolic blood pressure reportedly had a large effect (d = −0.9, 95% CI: −1.9, 0.2; d = −0.7, 95% CI: −1.36, −0.9) [37,58].

##### Effects of Continuous Positive Airway Pressure Versus Standard Care on Systolic Blood Pressure at Specific Times of Day

The daytime and nighttime systolic blood pressures reportedly showed a small effect size (d = −0.2, 95% CI: −0.57, 0.15; d = −0.2, 95% CI: −0.54, 0.17) to a large effect size (d = −0.8, 95% CI: −1.76, 0.14; d = −0.7, 95% CI: −1.67, 0.20), respectively [40,62]. The nighttime mean blood pressure showed a trivial effect size (d = −0.08, 95% CI: −0.44 to 0.27) [62]. Combining CPAP with telemedicine resulted in very large effects in the morning (d = 3.3, 95% CI: −2.9 to 9.4) and in the evening (d = −2.4, 95% CI: −7.2 to 2.4) [44]; it also resulted in a large effect when compared with medication (d = 0.8, 95% CI: −0.08 to 1.65) [61]. The effect on wake-mean systolic blood pressure was large (d = −1.64, 95% CI: −2.25 to −1.02) [49]. Similarly, the sleep mean systolic blood pressure effect was also large (d = −0.8, 95% CI: −1.28 to −0.29) (Table 3) [49].

##### Effects of Continuous Positive Airway Pressure Versus Usual Care on Diastolic Blood Pressure at Specific Times of Day

Again, a large effect (d = −0.6, 95% CI: −1.6, 0.3) was reported for nighttime [37]. The nighttime diastolic blood pressure at six weeks (d = −0.2, 95% CI: −0.42, 0.009) and twelve weeks (d = −0.1, 95% CI: −0.32, 0.11) showed small effect sizes [39]. Similarly, there was a small improvement at both six months (d = −0.1, 95% CI: −0.41, 0.21) and twelve months (d = −0.3, 95% CI: −0.02, 0.60) [60]. Uniquely, the mean 24 h diastolic blood pressure differs, with six weeks showing a large effect (d = −0.5, 95% CI: −0.68, −0.24), while twelve weeks shows a small effect (d = 0.2, 95% CI: −0.44, −0.01) (Table 3) [39].

A moderate effect size (d = −0.4, 95% CI: −1.07, 0.29) was reported for morning diastolic blood pressure, and a large effect size (d = 0.6, 95% CI: −0.22, 1.45) was reported for daytime diastolic blood pressure [45]. In contrast, a small effect (d = −0.3, 95% CI: −1.02, 0.34; d = −0.2, 95% CI: −0.92, 0.42) and moderate (d = −0.5, 95% CI: −1.15, 0.23) effects were reported in the afternoon [45] (Table 3).

Again, a study reported mean differences for daytime diastolic blood pressure (0.5, 95% CI: −0.9, −8.0) and nighttime diastolic blood pressure (0.3, 95% CI: −0.8, 1.5) [54]. Furthermore, a trivial (d = −0.08, 95% CI: −0.39, 0.23) to small improvement was observed at six and twelve months, respectively, for nighttime diastolic blood pressure [60]. The difference for nighttime mean blood pressure was 0.3 (95% CI: −1.0, 1.7), as well as for daytime mean blood pressure (0.3, 95% CI: −1.3, 1.8) [54].

The effect of nighttime dipping on mean blood pressure reportedly had a mean difference of −0.1 (95% CI: −1.2, −1.1) [54]. The 24 h daytime diastolic blood pressure showed a small effect (d = −0.3, 95% CI: −0.71, 0.14) [56]. In contrast, the 24 h nighttime diastolic blood pressure reportedly had a moderate effect size (d = −0.5, 95% CI: −0.96, −0.08; d = −0.5, 95% CI: −1.07, 0.15) (Table 3) [56,58].

##### Effects of Continuous Positive Airway Pressure Versus Standard Care on Diastolic Blood Pressure at Specific Times of Day

A small change was observed in daytime diastolic blood pressure, with an effect size of d = −0.2 (95% CI: −1.1, 0.7), and in nighttime diastolic blood pressure (d = −0.3, 95% CI: −1.2, 0.6) [37]. Similarly, there was a large effect (d = −0.6, 95% CI: −1.52, 0.31; d = 1.0, 95% CI: 0.07, 1.88) compared with medication [40,61]. However, the mean diastolic blood pressure shows a trivial effect size (d = −0.009, 95% CI: −0.45, 0.26) [62]. Viewed differently, there was a large effect (d = −0.6, 95% CI: −1.55, 0.28) for night-time diastolic blood pressure; however, the comparison was against medication, so the changes, hence the changes [40]. Combining CPAP with telemedicine resulted in a small effect (d = 0.1, 95% CI: −2.7, 3.0) in the morning and a trivial effect in the evening (d = −0.03, 95% CI: −2.6, 2.5) [44]. The effect on wake-mean diastolic blood pressure was large (d = −1.2, 95% CI: −1.77, −0.68) [49]. However, the effect on mean diastolic blood pressure during sleep was small (d = −0.3, 95% CI: −0.79, 0.12) [49]. The daytime diastolic blood pressure reportedly had a small effect size (d = −0.2, 95% CI: −0.55, 0.16) (Table 3) [62].

##### Effects of Continuous Positive Airway Pressure Versus Usual Care on 24 h Final Systolic Blood Pressure

The 24 h and final systolic blood pressure reportedly had a trivial effect (d = 0.04, 95% CI: −0.23 to 0.30; d = 0.09, 95% CI: −0.31 to 0.46; d = 0.04, 95% CI: −0.27 to 0.30) to a small effect (d = 0.3, 95% CI: −0.09 to 0.22; d = −0.2, 95% CI: −0.57 to 0.15), respectively (Table 3) [47,50,60,62].

##### Effects of Continuous Positive Airway Pressure Versus Usual Care on Changes in 24 h Mean Systolic Blood Pressure

The changes in 24 h mean systolic blood pressure reportedly have a small (d = 3.0, 95% CI: 2.21, 3.79) to moderate effect size (d = 0.4, 95% CI: 0.10, 0.64) [43,50,51]; in contrast, a small effect was observed at both six weeks (d = −0.2, 95% CI: −0.46, −0.03) and 12 weeks (d = −0.2, 95% CI: −0.38, 0.05) [39].

##### Effects of Continuous Positive Airway Pressure Versus Usual Care on Changes in 24 h Mean Diastolic Blood Pressure

The 24 h diastolic blood pressure showed a moderate effect (d = −0.5, 95% CI: −0.93 to −0.06) (Table 3) [56]. Furthermore, the mean 24 h diastolic blood pressure at six weeks (d = −0.2, 95% CI: −0.45, 0.02) and twelve weeks (d = −0.2, 95% CI: −0.44, −0.01) also showed small effect sizes, respectively (Table 3) [39].

##### Effects of Continuous Positive Airway Pressure Versus Standard Care on Changes in 24 h Mean Diastolic Blood Pressure

A trivial (d = −0.03, 95% CI: −0.36, 0.41) to small effect (d = −0.2, 95% CI: −0.39, 0.06; d = −0.2, 95% CI: −0.55, 0.17) and a large effect (d = −0.8, 95% CI: −1.72, 0.17; d = −1.0, 95% CI: −1.52, −0.48; d = −1.5, 95% CI: −1.45, −1.96; d = −0.8, 95% CI: −1.47, −0.17), as well as nighttime mean blood pressure (d = −0.7, 95% CI: −1.63, 0.23), were reported [40,47,49,51,58,62]. The daytime mean blood pressure also showed a large effect size (d = −0.7, 95% CI: −1.67, 0.20) [40]. There was a small improvement at both six (d = 0.1, 95% CI: −0.40, 0.21) and twelve months (d = −0.2, 95% CI: −0.53, 0.09) (Table 3) [60].

##### Effects of Continuous Positive Airway Pressure Versus Usual Care on Final Medication-Adjusted Clinic, Office, and Home Systolic Blood Pressure

Trivial effects were reported for final medication adjustment (d = 0.009, 95% CI: −0.25, 0.27) and clinic systolic blood pressure (d = 0.02, 95% CI: −0.37, 0.41) [47,50]. The effects on office systolic blood pressure ranged from small (d = −0.1, 95% CI: −0.34, 0.11) to moderate (d = −0.4, 95% CI: −0.78, −0.11) [29,36]. Uniquely, home systolic blood pressure reportedly showed a large effect (d = −0.7, 95% CI: −1.04, −0.34) (Table 3) [48].

##### Effects of Continuous Positive Airway Pressure Versus Usual Care on Final Medication-Adjusted Clinic, Office, and Home Diastolic Blood Pressure

A small effect size (d = −0.1, 95% CI: −0.49, 0.29) was reported for the clinic diastolic blood pressure [47]. The effect on office diastolic pressure was moderate (d = −0.5, 95% CI: −0.88, −0.20) [48]. The home diastolic blood pressure reportedly had a large effect (d = −0.9, 95% CI: −1.26, −0.53) (Table 3) [48].

##### Effects of Continuous Positive Airway Pressure Versus Standard Care on Peripheral and Central Systolic Blood Pressure

For peripheral pressure, effects ranged from trivial (d = 0.04, 95% CI: −0.87, 0.95) to large (d = −0.6, 95% CI: −0.33, 1.61); however, for the central effect, only small effects were reported (d = 0.2, 95% CI: −0.7, 1.1; d = 0.2, 95% CI: −0.7, 1.1; d = −0.2, 95% CI: −0.55, 0.21) (Table 3) [41,62].

##### Effects of Continuous Positive Airway Pressure Versus Standard Care on Peripheral and Central Diastolic Blood Pressure

Small effect sizes were reported for both peripheral (d = −0.3, 95% CI: −0.58, 1.61) and central diastolic blood pressure (d = −0.2, 95% CI: −0.68, 1.15; d = −0.2, 95% CI: −0.63, 0.14) (Table 3) [41,62].

## 4. Discussion

This is a structured narrative review of randomised controlled trials that synthesises and compares evidence on how CPAP intervention may help control blood pressure in patients with obstructive sleep apnoea. It compares these interventions with usual and standard care to determine which may influence blood pressure (systolic and diastolic), using only randomised controlled trials, with particular attention to the time of day, such as morning, afternoon, evening, nighttime (nocturnal), or daytime (diurnal).

Evidence from this study indicates that CPAP may be an effective intervention for reducing both systolic and diastolic blood pressure in patients with obstructive sleep apnoea. CPAP reportedly produced small to large effect sizes compared with usual care. Similarly, a large effect was observed when standard care (medication and telemedicine) was used. This suggests that the effects of standard care may not necessarily differ from those of CPAP. However, it is important to note that effect sizes from comparisons with pharmacological agents may not be methodologically equivalent to those from comparisons with no treatment or sham CPAP, and conflating these might inflate the apparent range of CPAP effects. It is also noteworthy that we did not observe any significant variation in outcomes when comparing CPAP with usual care and standard care (sham CPAP, no therapy, antihypertensive medication such as Valsartan, telemedicine support, nasal dilator strips (NDS), and acetazolamide), nor in their differences across studies. There was also evidence of sustained effects at 3, 6, and 12 months, although the magnitude was small. The shorter the duration of CPAP, the better the outcomes for both systolic and diastolic blood pressure; however, the effects tend to stabilise as the interventions continue for longer periods, although there is some variability at different times of the day.

Corroborating a similar study, the literature has shown that CPAP, compared to usual care, results in a reduction in both systolic and diastolic blood pressure [66]. Similarly, this study showed that the effect size for continuous airway pressure compared to usual care appears to be similar for both systolic and diastolic blood pressure, with both showing small to large effects. Furthermore, systolic blood pressure showed small to moderate effects, while diastolic blood pressure showed a large effect for continuous compared to usual care. CPAP reportedly had a large effect on both systolic and diastolic blood pressure over six to twelve months. Studies have shown that CPAP intervention for patients with obstructive sleep apnoea and a higher risk of hypertension over 6–12-month periods results in lowered systolic and diastolic blood pressure. Systematic review evidence has shown that peripheral systolic blood pressure is the most frequently studied; however, diastolic blood pressure reportedly has a larger effect [67]. This study supports these findings, as a small to moderate effect size was observed for both central and peripheral systolic blood pressure, while diastolic blood pressure showed a large effect size. Both systolic and diastolic blood pressures showed small to moderate effect sizes in the morning and afternoon, respectively. Similarly, research has shown that systolic and diastolic blood pressure exhibit daytime rhythms, with afternoon and morning measurements differing significantly; however, in clinical terms, both are often characterised by small to moderate effects [68,69].

When CPAP is compared to standard care, particularly medication, the effect size for systolic blood pressure ranges from trivial to large, while that for diastolic blood pressure appears to be large. Conversely, studies have shown that CPAP, compared to standard care, results in a small to moderate effect, but not a large one [70]. When CPAP is compared to standard care, particularly telemedicine, a large effect was reported in the morning and evening for systolic blood pressure; in contrast, a trivial effect was reported in the evening and a small effect in the morning for diastolic blood pressure. Evidence has shown that, compared to CPAP, telemedicine could potentially reduce blood pressure in both the morning and evening [71]. In contrast, some studies found that CPAP itself might have a strong effect on systolic blood pressure compared to standard care [44].

When CPAP was compared with standard care for 24 h mean blood pressure, a small to large effect was reported for systolic blood pressure, while diastolic blood pressure showed trivial to large effect sizes. Similarly, meta-analytic evidence indicated that CPAP, compared to standard care, resulted in a moderate effect on 24 h mean systolic and diastolic blood pressure [44]. Furthermore, within three to twelve months, a trivial to small effect was observed for systolic blood pressure, while diastolic blood pressure also showed a small effect size. This demonstrates a sustained effect of the intervention.

**Figure 3:**
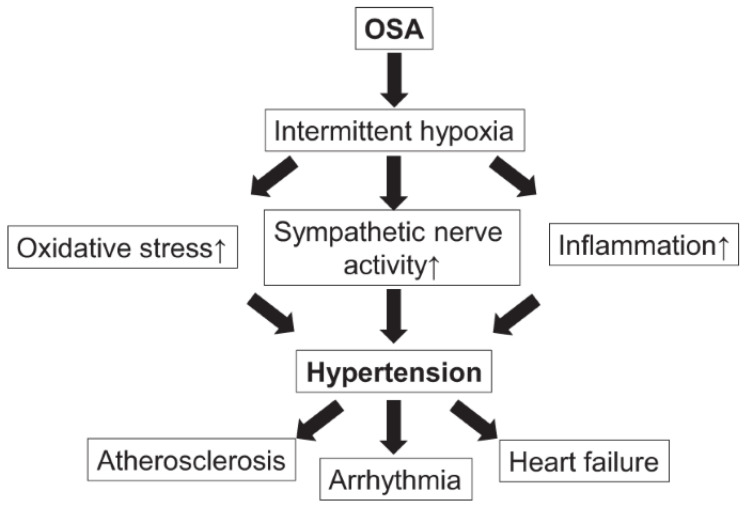
Various mechanisms of OSA-induced hypertension and cardiovascular diseases.

As shown in Figure 3, the probable mechanism by which CPAP reduces hypertension may involve a reduction in inflammatory responses, decreased sympathetic activation and oxidative stress, as well as improved endothelial function [39,60]. Evidence indicates that OSA may be associated with cardiovascular events such as hypertension [10]. Hypothetically, elevation of blood pressure might result from regulation of the sympathetic nervous system via both baroreceptors and chemoreceptors in the carotid artery [72]. Specifically, a reduction in arterial blood oxygen concentration may stimulate the carotid body, which then excites the afferent nerve to activate the respiratory centre in the hypothalamus, leading to increased hyperactivity of the efferent nervous system [10]. The increase in blood pressure is caused by heightened sympathetic nerve activity alongside weakened baroreceptor function [10]. However, OSA that persists for a longer period may lead to increased blood pressure not only at night but also during the daytime, possibly due to functional changes in baroreceptor and chemoreceptor activity [10]. Other hypotheses include the possible role of renin–angiotensin–aldosterone system elevation [73]. Evidence shows that intermittent hypoxia may increase plasma aldosterone levels by inducing the renin system and angiotensin II type 1 receptors, which subsequently results in increased blood pressure [53,74]. Furthermore, it has been established that oxidative stress may induce contraction of blood vessels secondary to endothelial dysfunction, leading to increased blood pressure. However, treatment with CPAP for OSA has been reported to improve vascular cell inflammation [75], which might be the probable operational mechanism of this intervention.

This review may be highly susceptible to bias due to its solo authorship. Further, there may be a lack of transparent methodology, although the author attempted to select high-quality evidence (RCTs). Additionally, the review presents a restricted perspective compared to collaborative work, as working alone is likely to limit critical analysis; there was no one to challenge assumptions, debate interpretations, or help identify research gaps. The distribution of study locations and gender may also be a concern, as males were the dominant population, and there was not a single study from Africa. It is also difficult to determine the effective dose of CPAP on outcomes due to unclear duration and, in most cases, the frequency of the interventions. Furthermore, searching only the first ten pages of Google Scholar for the grey literature may not be an adequate or reproducible approach. Our second objective was to determine the effective dose of CPAP on systolic and diastolic blood pressure outcomes. However, due to insufficient data, we were unable to address this objective; thus, it remains a major unresolved gap. It is also an established criterion that the minimum required CPAP adherence was typically at least four hours per night; however, it was not consistently reported in many of the eligible studies. The use of a pre-post correlation of r = 0.5 uniformly across all studies to calculate Cohen’s d may be a pragmatic assumption in the absence of individual-level data, but it is likely to introduce systematic error. Finally, it is important to acknowledge that the synthesis of results in this study involved heterogeneous samples with variations in OSA severity, hypertension status, CPAP adherence, treatment duration, and blood pressure measurement method. Therefore, caution should be exercised when using the outcomes of this study.

Although most of the study population in this review was men (63.3%), which is consistent with the existing literature indicating that OSA is more prevalent in men [9], a growing body of evidence identifies OSA as a condition with sex-specific variations, particularly regarding cardiovascular consequences and pathophysiology [76]. OSA is a sex-specific cardiovascular risk factor, especially as it is underdiagnosed in women [10]. OSA often remains undiagnosed and undertreated in women, partly because screening tools and clinical criteria have been developed mainly in male cohorts [10]. Furthermore, women with OSAS tend to present with more atypical symptoms, have a greater burden of cardiovascular comorbidities, and are at risk of being underdiagnosed [76].

Evidence from this study indicates that CPAP may be effective for hypertensive patients with co-existing obstructive sleep apnoea. However, this effect may vary at different times of day. Clinically, it may help provide cardiovascular protection, potentially reducing associated mortality and morbidity. Thus, CPAP could serve as an adjunct therapy for hypertensive patients with obstructive sleep apnoea. To support these findings, the literature has shown that OSA is associated with various chronic medical conditions, including cardiovascular diseases (e.g., hypertension), respiratory diseases (e.g., asthma and chronic obstructive pulmonary disease [COPD]), neurological diseases (e.g., memory loss, cognitive decline, anxiety, and depression), and metabolic disorders such as type 2 diabetes mellitus, among others [1,10].

## 5. Conclusions

CPAP may be associated with small and variable reductions in systolic and diastolic blood pressure in patients with obstructive sleep apnoea and hypertension, particularly in ambulatory or nighttime measurements. However, the evidence is heterogeneous, superiority over comparator interventions is not consistently demonstrated, and the optimal CPAP dose or adherence threshold remains unclear.

## Figures and Tables

**Table 1:** Quality ratings: Risk of bias within studies. Quality appraisal using the PEDro scale.

No	Author	Eligibility	Randomised Allocation	Concealed Allocation	Similarity at Baseline	Blinding of Participant	Blinding of Therapist	Blinding of Assessor	Dropout	Intention to Treat	Group Comparison	PMVD	Total Score (10)	Internal Validity (8)	Sub Scale (2)	Interpretation
1	[36]	Yes	Yes	Yes	Yes	Yes	No	Yes	Yes	Yes	Yes	Yes	9	7	2	Excellent
2	[37]	No	Yes	No	Yes	Yes	No	No	Yes	No	Yes	Yes	6	4	2	Good
3	[38]	Yes	Yes	No	Yes	No	No	No	No	No	Yes	Yes	4	2	2	Moderate
4	[39]	Yes	Yes	Yes	Yes	No	No	Yes	No	Yes	Yes	Yes	7	5	2	Good
5	[40]	No	Yes	No	Yes	No	No	No	No	Yes	Yes	Yes	5	3	2	Moderate
6	[41]	Yes	Yes	Yes	Yes	Yes	No	No	Yes	No	Yes	Yes	7	5	2	Good
7	[42]	Yes	Yes	Yes	No	No	No	Yes	Yes	Yes	Yes	Yes	7	5	2	Good
8	[43]	No	Yes	No	Yes	No	No	No	No	No	Yes	Yes	4	2	2	Moderate
9	[44]	Yes	Yes	No	Yes	No	No	No	No	Yes	Yes	Yes	5	3	2	Moderate
10	[45]	Yes	Yes	Yes	Yes	No	No	No	No	Yes	Yes	Yes	6	4	2	Good
11	[46]	Yes	Yes	Yes	Yes	No	No	Yes	Yes	No	Yes	Yes	7	5	2	Good
12	[47]	Yes	Yes	Yes	Yes	No	No	Yes	Yes	Yes	Yes	Yes	8	6	2	Good
13	[48]	No	Yes	No	Yes	No	No	No	Yes	Yes	Yes	Yes	6	4	2	Good
14	[49]	Yes	Yes	Yes	No	No	No	No	Yes	No	Yes	Yes	5	3	2	Moderate
15	[50]	Yes	Yes	No	Yes	No	No	No	Yes	No	Yes	Yes	5	3	2	Moderate
16	[51]	Yes	Yes	No	No	No	No	Yes	Yes	Yes	Yes	Yes	6	4	2	Good
17	[52]	No	Yes	No	No	Yes	No	No	No	No	Yes	Yes	4	2	2	Moderate
18	[53]	Yes	Yes	No	Yes	No	No	Yes	Yes	Yes	Yes	Yes	7	5	2	Good
19	[54]	Yes	Yes	No	Yes	Yes	Yes	No	No	No	Yes	Yes	6	4	2	Good
20	[55]	Yes	Yes	Yes	Yes	No	No	Yes	Yes	Yes	Yes	Yes	8	6	2	Good
21	[56]	Yes	Yes	No	Yes	No	No	No	Yes	Yes	Yes	Yes	6	4	2	Good
22	[57]	No	Yes	No	Yes	No	No	Yes	Yes	Yes	Yes	Yes	7	5	2	Good
23	[58]	Yes	Yes	No	Yes	Yes	No	No	No	No	Yes	Yes	5	3	2	Moderate
24	[59]	Yes	Yes	Yes	No	No	No	Yes	Yes	No	Yes	Yes	6	4	2	Good
25	[60]	Yes	Yes	No	No	No	No	Yes	No	No	Yes	Yes	4	2	2	Moderate
26	[61]	Yes	Yes	No	Yes	No	No	Yes	No	No	Yes	Yes	5	3	2	Moderate
27	[62]	Yes	Yes	Yes	Yes	No	No	No	Yes	Yes	Yes	Yes	7	5	2	Good
28	[63]	Yes	Yes	Yes	Yes	No	No	Yes	No	Yes	Yes	Yes	7	5	2	Good

PMVD = Point measures and variability data. Note: Each item was scored either Yes = 1 or No = 0. Items 2–11 are summed for a PEDro total score. The sum of items 2–9 yields the internal validity subscale score, while the sum of items 10 and 11 yields the statistical reporting subscale score. The PEDro total score was rated as 0–3 = poor, 4–5 moderate, 6–8 good, and 9–10 = excellent.

**Table 2:** Demographic characteristics.

					Study Population			
No	Author(s)	Age in Years/Range/M(SD)	Gender (#, % of Male and #, % of Females)	Study Population Category/Study Design/N	Intervention Group, Name	Control Group, Name	Place of Intervention (Community, Nursing Home, Hospital, etc.)	Country of Study
1	[36]	CPAP (therapeutic) 55.3 ± 9.6 CPAP (subtherapeutic) 58.0 ± 97.0	Males *n* = 41, 60.3% Female *n* = 27, 39.7%	OSA/RCT/38	CPAP (therapeutic)	CPAP (subtherapeutic)	Hospital/home	Spain
2	[37]	Control: 50 ± 10 OSA: 51 ± 13	Males *n* = 20, 95.2% Females *n* = 1, 4.7%	OSA patients & healthy subjects/RCT/21	CPAP	Sham CPAP (healthy subjects)	Hospital	Unclear
3	[38]	55.5	Males (*n* = 9, 69.2%) Females (*n* = 4, 30.8%)	OSA/RCT/13	CPAP	NO therapy (usual care)	Hospital	Canada
4	[39]	52.4 (SD 10.5)	Males (*n* = 277, 81.5%) Females (*n* = 63, 18.5%)	OSA/RCT/340	CPAP	Sham CPAP	Hospital	Spain
5	[40]	57–68	Males (*n* = 19, 82.6%) Females (*n* = 4, 17.4%)	OSA/RCT/23	CPAP	Valsartan	Hospital	France
6	[41]	23–59	Females *n* = 3, 25% Males *n* = 9, 75%	OSA patient/RCT/12	CPAP	APAP	Home	Brazil
7	[42]	56 ± 10	Males (*n* = 167, 46.6%) Females (*n* = 191, 53.4%)	OSA/RCT/358	CPAP	Conservative group	Hospitals	Spain
8	[43]	57.2 ± 1.7	Males (*n* = 52, 96.4%) Females (*n* = 3, 5.6%)	OSA/RCT/55	CPAP	Control	Home	Australia
9	[44]	63 ± 9	Males (*n* = 89, 83.2%) Females (*n* = 18, 16.8%)	OSA/RCT/107	CPAP + Standard Care	CPAP + Telemedicine	Home	France
10	[45]	49.1 ± 13.6	Males (*n* = 26, 86.7%) Females (*n* = 4, 13.3%)	OSA/RCT/30	CPAP	Sham CPAP	Hospital	Australia
11	[46]	Control: 62.7 ± 6.7 CPAP: 62.0 ± 6.8	Males (*n* = 60, 82.2%) Females (*n* = 13, 17.8%)	OSA/RCT/73	CPAP	Control	Hospital	China
12	[47]	60.5 (8.2)	Males (*n* = 47, 39.8%) Females (*n* = 70, 46.6%)	OSA/RCT/117	CPAP	Control	Hospital	Brazil
13	[48]	CPAP group 62.8 (7.9) Withdrawal group 62.8 (9.0)	Males (*n* = 126, 84.5%) Females (*n* = 23, 15.5%)	OSA/RCT/149	CPAP	Withdraw CPAP	Home	Switzerland & United Kingdom
14	[49]	52 ± 9	Males (*n* = 69, 81.2%) Females (*n* = 16, 18.8%)	OSA/RCT/85	Perindopril 10 mg CPAP (AM Dose)	Perindopril 10 mg CPAP (PM Dose)	Hospital	Australia
15	[50]	Intervention: 69.7 ± 10.4 Control: 70.2 ± 10.1	Males (*n* = 219, 97.3%) Females (*n* = 6, 2.7%)	OSA/RCT/225	CPAP	Control	Home	USA
16	[51]	57.1 ± 10.1	Females (*n* = 307, 100%)	OSA/RCT/307	CPAP	Conservative treatment	Hospital	Spain
17	[52]	60 ± 10	Males (*n* = 48, 77.4%) Females (*n* = 14, 22.6%)	OSA/RCT/62	CPAP	Sham CPAP	Hospital	France
18	[53]	55.0 ± 9.6	Males (*n* = 52, 81.3%) Females (*n* = 12, 38.7%)	OSA/RCT/64	CPAP	Control	Hospital	China
19	[54]	56 ± 11	Males (*n* = 26, 81.25%) Females (*n* = 6, 18.75%)	OSA/RCT/32	CPAP	Sham CPAP	Hospital	Spain
20	[55]	64 ± 7	Males (*n* = 13, 100%)	OSA/Hypertension/RCT/13	CPAP	CPAP + Acetazolamide Acetazolamide	Hospital	Sweden
21	[56]	59.9 (9.8)	Males (*n* = 57, 63.3%) Females (*n* = 33, 36.7%)	CPAP/RCT/90	CPAP	Control	Hospital	China
22	[57]	60.6 (8.0)	Males (*n* = 46, 39.7%) Female (*n* = 70, 60.3%)	OSA/RCT/116	CPAP	Control	Hospital	Brazil
23	[58]	Intervention: 45.9 ± 9.8 Control: 50.4 ± 10.0	Males (*n* = 51, 85%) Females (*n* = 9, 15%)	OSA/RCT/60	CPAP	Sham CPAP	Hospital/Home	China
24	[59]	51 ± 8	Males (*n* = 71, 77.2%) Females (*n* = 21, 22.8%)	OSA/RCT/92	CPAP	Control	Hospital	China
25	[60]	63.8 (7.3)	Males (*n* = 110, 65.1%) Females (*n* = 59, 34.9%)	OSA/RCT/169	CPAP	No CPAP	Hospital	USA
26	[61]	36.5± 5.0	Females (*n* = 48, 100%)	OSA/RCT/48	CPAP	NDS	Home	Canada
27	[62]	55(49–60)	Males (*n* = 92, 63.4%) Females (*n* = 53, 36.6%)	OSA/RCT/145	CPAP	NDS	Home	Brazil
28	[63]	Control 65(9) Intervention 63(9)	Females (*n* = 33, 33%) Males (*n* = 67, 67%)	OSA/RCT/100	CPAP	No CPAP	Hospital	Sweden
			Total males (*n* = 1863, 63.3%) Total females (*n* = 1081, 36.7%)	Total = 2944				

OSA: Obstructive Sleep Apnoea; CPAP: Continuous Positive Airway Pressure; RCT: Randomised Controlled Trial; APAP: Positive Airway Pressure; USA: United States of America; NDS: Nasal Dilator Strip.

**Table 3:** Effects of continuous positive airway pressure on blood pressure of patients with obstructive sleep apnoea.

		Dose					
No	Author/Year	Duration	Frequency	Course	Intervention Loads	Measures	Effect Sizes (Between-Group Effects (Using Pretest-Posttest SD)
1	[36]	24 h	Unclear	Unclear		Sphygmomanometer	24 h Mean Blood Pressure Cohen’s d = −0.2, 95% CI −0.69, 0.28 * 24 h Systolic Blood Pressure Cohen’s d = 0.06, 95% CI −0.43, 0.54 * 24 h Diastolic Blood Pressure Cohen’s d = 0.07, 95% CI −0.41, 0.56 * Daytime Blood Pressure Cohen’s d = 0.09, 95% CI −0.39, 0.57* Nighttime Blood Pressure Cohen’s d = 0.02, 95% CI −0.46, 0.50 *
2	[37]	Unclear	Unclear	12 weeks	NA	Echocardiogram ABPM	Daytime systolic blood Pressure Cohen’s d = −0.9, 95% CI −1.9, 0.2 * Daytime Diastolic Blood Pressure Cohen’s d = −0.2, 95% CI −1.1, 0.7 * Nighttime Systolic Blood Pressure Cohen’s d = −0.6, 95% CI −1.6, 0.3 * Nighttime Diastolic Blood Pressure Cohen’s d = −0.3, 95% CI −1.2, 0.6 *
3	[38]	Unclear	Unclear	4 weeks	NA	Sphygmomanometer	Systolic Blood Pressure Difference: change with–change without CPAP −3.60 (95% CI −18.45, 11.25 *, *p* = 0.50) Diastolic Blood Pressure Difference: change with–change without CPAP −0.70 (−8.53, 7.13 *, *p* = 0.84)
4	[39]	24 h	Not specified	12 weeks	NA	Cuff measurement	Daytime Systolic Blood Pressure (week 6) Cohen’s d = −0.2, 95% CI −0.46, −0.03 * Daytime Systolic Blood Pressure (week 12) Cohen’s d = −0.2,95% CI −0.38, 0.05 * Daytime Diastolic Blood Pressure (week 6) Cohen’s d = −0.2, 95% CI −0.44, −0.01 * Daytime Diastolic Blood Pressure (week 12) Cohen’s d = −0.2, 95% CI −0.44, −0.007 * Daytime Mean Blood Pressure (week 6) Cohen’s d = −0.1, 95% CI −0.32, 0.11 * Daytime Mean Blood Pressure (week 12) Cohen’s d = −0.2, 95% CI −0.21, 0.21 * Nighttime Systolic Blood Pressure (week 6) Cohen’s d = −0.4, 95% CI −0.57, −0.13 * Nighttime Systolic Blood Pressure (week 12) Cohen’s d = −0.3, 95% CI −0.50, 0.06 * Nighttime Diastolic Blood Pressure (week 6) Cohen’s d = −0.2, 95% CI −0.42, 0.009 * Nighttime Diastolic Blood Pressure (week 12) Cohen’s d = −0.1, 95% CI −0.32, 0.11 * Mean 24 h Systolic Blood Pressure (week 6) Cohen’s d = −0.2, 95% CI −0.46, −0.03 * Mean 24 h Systolic Blood Pressure (week 12) Cohen’s d = −0.2, 95% CI −0.38, 0.05 * Mean 24 h Diastolic Blood Pressure (week 6) Cohen’s d = −0.2, 95% CI −0.45, −0.02 * Mean 24 h Diastolic Blood Pressure (week 12) Cohen’s d = −0.2, 95% CI −0.44, −0.01 * Mean 24 h BP (week 6) Cohen’s d = −0.5, 95% CI −0.68, −0.24 * Mean 24 h BP (week 12) Cohen’s d = −0.2, 95% CI −0.44, −0.01 *
5	[40]	24 h	Unclear	8 Weeks	NA	ABPM	24 h Systolic Blood Pressure Cohen’s d = −0.8, 95% CI −0.72, 2.27 * 24 h Diastolic Blood Pressure Cohen’s d = −0.7, 95% CI −1.60, 0.25 * 24 h Mean Blood Pressure Cohen’s d = −0.8, 95% CI −1.72, 0.17 * Daytime Systolic Blood Pressure Cohen’s d = −0.8, 95% CI −1.76, 0.14 * Daytime Diastolic Blood Pressure Cohen’s d = −0.6, 95% CI −1.52, 0.31 * Daytime Mean Blood Pressure Cohen’s d = −0.7, 95% CI −1.67, 0.20 * Nighttime Systolic Blood Pressure Cohen’s d = −0.7, 95% CI −1.67, 0.20 * Nighttime Diastolic Blood Pressure Cohen’s d = −0.6, 95% CI −1.55, 0.28 * Nighttime Mean Blood Pressure Cohen’s d = −0.7, 95% CI −1.63, 0.23 *
6	[41]	Unclear	Unclear	4 nights		Central Blood Pressure/or Aix measurement	Peripheral Systolic Blood Pressure Cohen’s d = 0.04, 95% CI −0.87, 0.95 * Peripheral Diastolic Blood Pressure Cohen’s d = 0, 95% CI −0.91, 0.91 * Peripheral Mean Blood Pressure Cohen’s d = 0.1, 95% CI −0.8, 1.0 Central Systolic Blood Pressure Cohen’s d = 0.2, 95% CI −0.7, 1.1 * Central Diastolic Blood Pressure Cohen’s d = −0.01, 95% CI −0.92, 0.89 *
7	[42]	Unclear	Unclear	52 weeks	NA	Sphygmomanometer	Changes in Systolic Blood Pressure Cohen’s d = 0.08, 95% CI −0.13, 0.29 * Changes in Systolic Blood Pressure (6 months) Cohen’s d = 0.02, 95% CI −0.19, 0.22 * Changes in Systolic Blood Pressure (12 months) Cohen’s d = 0.1, 95% CI −0.07, 0.35 * Changes in Diastolic Blood Pressure Cohen’s d = −0.2, 95% CI −0.37, 0.04 * Changes in Diastolic Blood Pressure (6 months) Cohen’s d = 0.1, 95% CI −0.09, 0.33 * Changes in Diastolic Blood Pressure (12 months) Cohen’s d = 0.1, 95% CI −0.07, 0.35 *
8	[43]	Unclear	Unclear	12 weeks	NA	Sphygmomanometer	Mean Blood Pressure Cohen’s d = 3.0, 95% CI 2.21, 3.79 *
9	[44]	Unclear	3 days	16 weeks	NA	Sphygmomanometer	CPAP + Telemedicine Morning Systolic Blood Pressure Mean changes 3.3 (95% CI −2.9; 9.4 *, *p* = 0.49) Evening Systolic Blood Pressure Mean changes −2.4 (95% CI −7.2; 2.4 *, *p* = 0.22) CPAP + Standard care Morning Diastolic Blood Pressure Mean changes 0.1 (95% CI −2.7; 3.0, *p* = 0.90) Evening Diastolic Blood Pressure Mean changes −0.03 (95% CI −2.6; 2.5 *, *p* = 0.88)
10	[45]	Unclear	Unclear	8 weeks	NA	Sphygmomanometer	Peripheral Blood Pressure Systolic (Afternoon) Cohen’s d = −0.4, 95% CI −1.11, 0.26 * Systolic (Afternoon) Cohen’s d = −0.2, 95% CI −0.85, 0.48 * Diastolic (Morning) Cohen’s d = −0.4, 95% CI −1.07, 0.29 * Diastolic (Afternoon) Cohen’s d = −0.3, 95% CI −1.02, 0.34 * Central Blood Pressure Systolic (Morning) Cohen’s d = −0.2, 95% CI −0.89, 0.45 * Systolic (Afternoon) Cohen’s d = −0.4, 95% CI −1.04, 0.32 * Diastolic (Morning) Cohen’s d = −0.2, 95% CI −0.92, 0.42 * Diastolic (Afternoon) Cohen’s d = −0.5, 95% CI −1.15, 0.23 *
11	[46]	Unclear	Unclear	144 weeks	NA	Sphygmomanometer	Systolic Blood Pressure Cohen’s d = −0.2, 95% CI −0.64, 0.30 * Diastolic Blood Pressure Cohen’s d = 0.1, 95% CI −0.36, 0.57 *
12	[47]	Unclear	Unclear	24 weeks	NA	ABPM	Clinic Systolic Blood Pressure Cohen’s d = 0.02, 95% CI −0.37, 0.41 * 24 h Systolic Blood Pressure Cohen’s d = 0.09, 95% CI −0.31, 0.46 * Daytime Systolic Blood Pressure Cohen’s d = 0.04, 95% CI −0.35, 0.42 * Nighttime Systolic Blood Pressure Cohen’s d = −0.03, 95% CI −0.42, 0.35 * Clinic Diastolic Blood Pressure Cohen’s d = −0.1, 95% CI −0.49, 0.29 * 24 h Diastolic Blood Pressure Cohen’s d = −0.03, 95% CI −0.36, 0.41 * Daytime Diastolic Blood Pressure Cohen’s d = 0.008, 95% CI −0.38, 0.40 * Nighttime Diastolic Blood Pressure Cohen’s d = −0.03, 95% CI −0.42, 0.35 *
13	[48]	Unclear	Daily	2 weeks	NA	Validated standard digital automatic monitor	Office Systolic Blood Pressure Cohen’s d = −0.4, 95% CI −0.78, −0.11 * Office Diastolic Blood Pressure Cohen’s d = −0.5, 95% CI −0.88, −0.20 * Home Systolic Blood Pressure Cohen’s d = −0.7, 95% CI −1.04, −0.34 * Home Diastolic Blood Pressure Cohen’s d = −0.9. 95% CI −1.26, −0.53 *
14	[49]	Unclear	Unclear	8 weeks	NA	ABPM	Wake-Mean Systolic Blood Pressure Cohen’s d = −1.64, 95% CI −2.25, −1.02 * Sleep Mean Cohen’s d = −0.8, 95% CI −1.28, −0.29 * 24 h Mean Systolic Blood Pressure Cohen’s d = −1.5, 95% CI −1.45, 1.96 * Wake-Mean Diastolic Blood Pressure Cohen’s d = −1.2, 95% CI −1.77, −0.68 * Sleep Mean Diastolic Blood Pressure Cohen’s d = −0.3, 95% CI −0.79, 0.12 * 24 h Mean Diastolic Blood Pressure Cohen’s d = −1.0, 95% CI −1.52, −0.48 *
15	[50]	Unclear	unclear	52 weeks	NA	ABPM	Final Mean 24 h Systolic Blood Pressure Cohen’s d = 0.04, 95% CI −0.23, 0.30 *
16	[51]	Unclear	Unclear	12 weeks	NA	Sphygmomanometer	Systolic Blood Pressure Cohen’s d = −0.1, 95% CI −0.34, 0.11 * Diastolic Blood Pressure Cohen’s d = −0.2, 95% CI −0.41, 0.04 * Mean Blood Pressure Cohen’s d = −0.2, 95% CI −0.39, 0.06 *
17	[52]	Daily	Every 15 min	12 weeks	180 min	ABPM/Sphygmomanometer	Data needs conversion from Median to Mean & from IQR to SD
18	[53]	NA	NA	12 weeks	NA	Dinamap	Systolic Blood Pressure Cohen’s d = −0.6, 95% CI −1.17, −0.11 * Diastolic Blood Pressure Cohen’s d = −1.4, 95% CI −2.08, −0.79 *
19	[54]	Unclear	Unclear	12 weeks	NA	ABPM	Daytime Systolic Blood Pressure Mean difference −0.1 (95% CI −2.4, 2.1 *) Daytime Diastolic Blood Pressure Mean difference 0.5 (95% CI −0.9, −8.0) Daytime Mean Blood Pressure Mean difference 0.3 (95% CI −1.3, 1.8 *) Nighttime Diastolic Blood Pressure Mean difference 0.3 (95% CI −0.8, 1.5 *) Nighttime Mean Blood Pressure Mean difference 0.3 (95% CI −1.0, 1.7 *) Nighttime dipping Mean Blood Pressure Mean difference −0.1 (95% CI −1.2–1.1 *)
20	[55]	Unclear	NA	4 weeks	NA	Not specified	There was a reduction in mean arterial pressure with Acetazolamide alone and Acetazolamide plus CPAP, but not with CPAP alone.
21	[56]	24 h	NA	NA	NA	ABPM	24 h Systolic Blood Pressure Cohen’s d = −0.5, 95% CI, −0.98, −0.11 * 24 h Daytime Systolic Blood Pressure Cohen’s d = −0.2, 95% CI −0.59, 0.25 * 24 h Nighttime Systolic Blood Pressure Cohen’s d = −0.8, 95% CI −1.27, −0.35 * 24 h Diastolic Blood Pressure Cohen’s d = −0.5, 95% CI −0.93, −0.06 * 24 h Daytime Diastolic Blood Pressure Cohen’s d = −0.3, 95% CI −0.71, 0.14 * 24 h Nighttime Diastolic Blood Pressure Cohen’s d = −0.5, 95% CI −0.96, −0.08 *
22	[57]	Unclear	Unclear	24 weeks	NA	Not specified	Systolic Blood Pressure during cf-PWV Cohen’s d = −0.004, 95% CI −0.37, 0.36 *
23	[58]	24 h	NA	12 weeks	NA	Automated Oscillometric Blood Pressure Monitor	24 h Systolic Blood Pressure Cohen’s d = −0.7, 95% CI −1.35, −0.08 * 24 h Diastolic Blood Pressure Cohen’s d = −0.8, 95% CI −1.47, −0.17 * Daytime Systolic Blood Pressure Cohen’s d = −0.6, 95% CI −1.24, 0.01 * Daytime Diastolic Blood Pressure Cohen’s d = −0.7, 95% CI −1.36, −0.9 * Nighttime Systolic Blood Pressure Cohen’s d = −0.3, 95% CI −0.85, 0.34 * Nighttime Diastolic Blood Pressure Cohen’s d = −0.5, 95% CI −1.07, 0.15 *
24	[59]	24 h	Unclear	8 weeks	NA	ABPM	The CPAP group showed a significant reduction in 24 h Systolic Blood pressure (intergroup difference −4.4 mmHg, 95% CI −8.7 to −0.1 *; *p* = 0.046), 24 h Diastolic Blood pressure (−2.9 mmHg, 95% CI −5.5 to −0.2 *; *p* = 0.032), Daytime Systolic Blood Pressure (−5.4 mmHg, 95% CI −9.7 to −1.0; *p* = 0.016), and daytime DBP (−3.4 mmHg, 95% CI −6.1 to −0.8 *; *p* = 0.012).
25	[60]	24 h	Unclear	52 weeks	NA	ABPM	24 h Systolic Blood Pressure (6 months) Cohen’s d = 0.04, 95% CI −0.27, 0.35 * 24 h Systolic Blood Pressure (12 months) Cohen’s d = −0.09, 95% CI −0.40, 0.22 * Daytime Systolic Blood Pressure (6 months) Cohen’s d = 0.07, 95% CI −0.24, 0.38 * Daytime Systolic Blood Pressure (12 months) Cohen’s d = 0, 95% CI −0.31, 0.31 * Nighttime Systolic Blood Pressure (6 months) Cohen’s d = −0.07, 95% CI −0.38, 0.23 * Nighttime Systolic Blood Pressure (12 months) Cohen’s d = −0.2, 95% CI −0.49, 0.13 * 24 h Diastolic Blood Pressure (6 months) Cohen’s d = 0.1, 95% CI −0.40, 0.21 * 24 h Diastolic Blood Pressure (12 months) Cohen’s d = −0.2, 95% CI −0.53, 0.09 * Daytime Diastolic Blood Pressure (6 months) Cohen’s d = −0.1, 95% CI −0.41, 0.21 * Daytime Diastolic Blood Pressure (12 months) Cohen’s d = 0.3, 95% CI −0.02, 0.60 * Nighttime Diastolic Blood Pressure (6 months) Cohen’s d = −0.08, 95% CI −0.39, 0.23 * Nighttime Diastolic Blood Pressure (12 months) Cohen’s d = −0.2, 95% CI −0.49, 0.13 *
26	[61]	24 h	Day (30-min) & Night (1-h)	12 weeks	NA	Unspecified	24 h Systolic Blood Pressure Cohen’s d = 0.7, 95% CI −0.15, 1.55 * 24 h Diastolic Blood Pressure Cohen’s d = 0.8, 95% CI −0.09, 1.63 * Daytime Systolic Blood Pressure Cohen’s d = 0.6, 95% CI −0.22, 1.45 * Daytime Diastolic Blood Pressure Cohen’s d = 0.6, 95% CI −0.22, 1.45 * Daytime Mean Arterial Pressure Cohen’s d = 0.6, 95% CI −0.21, 1.47 * Nighttime Systolic Blood Pressure Cohen’s d = 0.8, 95% CI −0.08, 1.65 * Nighttime Diastolic Blood Pressure Cohen’s d = 1.0, 95% CI 0.07, 1.88 *
27	[62]	24 h	Unclear	6 months	NA	ABPM	Systolic Blood Pressure Cohen’s d = −1.0, 95% CI −1.38, −0.57 * Diastolic Blood Pressure Cohen’s d = −0.5, 95% CI −0.88, −0.14 * 24 h Systolic Blood Pressure Cohen’s d = −0.2, 95% CI −0.57, 0.15 * 24 h Diastolic Blood Pressure Cohen’s d = −0.2, 95% CI −0.55, 0.17 * Mean Blood Pressure Cohen’s d = −0.09, 95% CI −0.45, 0.26 * Daytime Systolic Blood Pressure Cohen’s d = −0.2, 95% CI −0.57, 0.15 * Daytime Diastolic Blood Pressure Cohen’s d = −0.2, 95% CI −0.55, 0.16 * Daytime Mean Blood Pressure Cohen’s d = −0.09, 95% CI −0.45, 0.26 * Nighttime Systolic Blood Pressure Cohen’s d = −0.2, 95% CI −0.54, 0.17 * Nighttime Mean Blood Pressure Cohen’s d = −0.08, 95% CI −0.44, 0.27 * Central Systolic Blood Pressure Cohen’s d = −0.2, 95% CI −0.55, 0.21 * Central Diastolic Blood Pressure Cohen’s d = −0.2, 95% CI −0.63, 0.14 *
28	[63]	24 h	30 min	1 week	720 min	SphygmoCor	42 h Systolic Blood Pressure Cohen’s d = 0.2, 95% CI −0.17, 0.63 * 24 h Diastolic Blood Pressure Cohen’s d = 0.2, 95% CI −0.17, 0.63 *

ABPM: Ambulatory Blood Pressure Monitoring; NA: Not Available; h: Hour; cf-PWV, Carotid-Femoral Pulse Wave Velocity; CI: Confidence Interval; *: Significant; DBP: Diastolic Blood Pressure.

## Data Availability

No new data were created or analysed in this study.
